# Point-of-Care Transcranial Doppler Sonography at the Intensive Care Unit—A Practical Review of the Fundamentals

**DOI:** 10.3390/jcm15041630

**Published:** 2026-02-20

**Authors:** Péter Siró, Zsófia Fülesdi, Csilla Molnár, Róbert Almási, László Csiba, Béla Fülesdi

**Affiliations:** 1 Department of Anesthesiology and Intensive Care, Faculty of Medicine, University of Debrecen, 4028 Debrecen, Hungary; 2 Department of Radiology, Faculty of Medicine, University of Debrecen, 4028 Debrecen, Hungary; 3 Department of Anesthesiology and Intensive Care, Faculty of Medicine, University of Pécs, 7624 Pécs, Hungary; 4 Department of Neurology, Faculty of Medicine, University of Debrecen, 4028 Debrecen, Hungary

**Keywords:** transcranial Doppler sonography, cerebral blood flow velocity, critical care

## Abstract

Point-of-care ultrasonography (POCUS) has become an integral part of intensive and emergency care. Despite the widespread use and availability of multipurpose ultrasound devices, the regular assessment of intracranial circulatory conditions has not become a part of daily routine in multidisciplinary intensive care units. This brief narrative review aims to summarize the fundamental knowledge about the transcranial Doppler technique and the most significant clinical areas in which the method can provide valuable assistance in daily diagnostic and therapeutic decision-making. The authors searched the PubMed database for reviews, systematic reviews, and meta-analyses using the keywords “transcranial Doppler sonography; critical care; cerebral vasospasm; brain death diagnosis; non-invasive intracranial pressure monitoring”. We conclude that TCD is a simple, yet skilled, bedside method for assessing intracranial circulation. In everyday practice, it can be used to support clinical decision-making primarily in the areas of intracranial pressure monitoring, diagnosis and follow-up of cerebral vasospasm, and diagnosis of cerebral circulatory arrest. The study of cerebral hemodynamics should be an integral part of the increasingly widespread bedside ultrasound diagnostics in intensive care.

## 1. Introduction

Point-of-care ultrasonography (POCUS) has become an integral part of intensive and emergency care in recent decades. It has been shown to be useful for hemodynamic monitoring and guiding fluid therapy [1], for diagnosis of pathologies of the lung and the abdominal organs [2,3,4,5], and it also serves as a useful tool in perioperative care [6]. As a consequence, it has become an important, integral part of the residency training programmes [7].

Although the widespread use and availability of modern ultrasound devices would make it possible, the regular assessment of intracranial circulatory conditions has not been widely used, except in neurointensive care units. It is an undeniable fact that mastering the transcranial Doppler method requires specific competencies, but in our opinion, the basic examination can be a useful additional part of the daily activities of general intensive care units. In a recent consensus document, basic and basic plus skills are listed for the general intensivists [8]. These basic skills include the knowledge of Doppler and echo-colour-Doppler parameters (depths, velocities, and power) and the assessment of the main Willisian vessels, as well as the internal carotid artery.

This simple review aims to summarize basic knowledge of the transcranial Doppler technique and the most important clinical areas in which the method can provide useful assistance in the everyday work of the intensivist. This narrative review article intentionally focuses on providing knowledge about practical implementation and areas of application that occur in an emergency or intensive care unit setting. The authors searched the PubMed database for reviews, systematic reviews, and meta-analyses using the keywords “transcranial Doppler sonography; critical care; cerebral vasospasm; brain death diagnosis; non-invasive intracranial pressure monitoring”. The description of the practical conduct of the transcranial Doppler and duplex sonography also includes the several decades of experience of the authors using this technique.

### 1.1. Technique of Performing Transcranial Doppler/Duplex Investigations

Transcranial Doppler/duplex investigations should usually be performed with the patients in the supine position. It is recommended that the examiner position himself at the patient’s head and fix his forearm on the bed. There is a technical reason for properly fixing the forearm: when finding the acoustic window, fine movement of the transducer position can be achieved primarily by moving the wrist, and this also ensures that the transducer position is stable. This is especially important if the acoustic window (the section of bone suitable for the passage and return of ultrasound) is very narrow. Positioning is important for several reasons: in the supine patient, the position of the arteriovenous pressure transfer (the zero point of intracranial pressure in a healthy person) is roughly at the height of the clivus.

**Figure 1:**
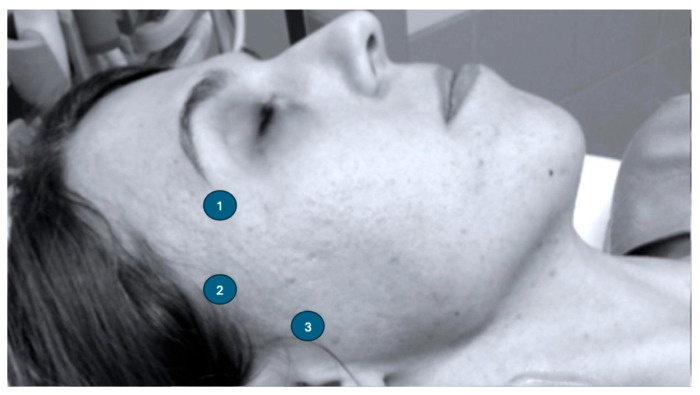
Typical sites for insonation of circle of Willis through the temporal window.

There are different windows on the skull that allow the assessment of the cerebral arteries: temporal, suboccipital and transorbital. Among them, the most widely used is the temporal window, as it allows for the approach to different arterial segments of the circle of Willis. The assessment of the intracranial segments of the vertebral arteries and the basilar artery is possible through the suboccipital window (foramen magnum). The registration of transcranial Doppler through the temporal bone is possible using three potential acoustic windows (Figure 1):

1. The anterior temporal window is located above the lateral canthus and can be easily identified as a depression in the bone when palpated with the fingertip. Using this temporal window, the transducer should be positioned so that the ultrasound beam is directed slightly posteriorly, towards the imaginary midline of the skull base.
2. The medial temporal window can be identified as a depression in the temporal bone above the junction of the head of the mandible with the zygomatic arch. This is the most commonly used temporal window. If this temporal window is chosen for measurement, the transducer position should be perpendicular to the bone surface.
3. The posterior temporal window is located anterior to and above the tragus. When examining in this position, the transducer should be tilted so that the ultrasound beam is positioned frontally and slightly upward.

There are basically two technical options for the assessment of cerebral circulation using the transcranial Doppler technique:

- Transcranial Doppler sonography is typically performed using a pulsed-wave probe with a frequency of 1–2 MHz. It only allows the measurement of the cerebral blood flow velocities in the different parts of the circle of Willis. This is the modality that allows bilateral continuous monitoring of the cerebral blood flow during surgical interventions and may also be part of the multimodal neuromonitoring in the intensive care unit. However, it is an independent device that can only be used for this purpose, given that angle correction of the ultrasound beam is not possible at the site of the assessed arterial segment. Furthermore, the headset that is used for fixing the transcranial Doppler probe may not ensure stable continuous monitoring in all cases, and thus, repositioning may be necessary.
- The most widely used technique for assessing the cerebral circulation is nowadays the use of transcranial colour-coded duplex sonography (TCCD). This technique usually uses a multifrequency transducer with 1.5–3 MHz spectral capability. In the majority of the multipurpose ultrasound devices used in the intensive care units, the same probe is used for transthoracic echocardiography. Duplex ultrasonography combines the B-mode (brightness mode) with the colour pulsed wave technique. This allows visual representation of the different segments of the circle of Willis, and by placing the pulsed wave cursor into the arterial segment, the measurement of the blood flow velocities in the different arteries is also feasible. A further advantage of the technique is that angle correction of the ultrasound beam can be performed on the visual of the vessel, which makes the blood flow velocity measurements more precise.

Since the duplex ultrasound examination technique is most available in multidisciplinary intensive care units, we will focus on the practical presentation of examinations performed with this technique.

**Figure 2:**
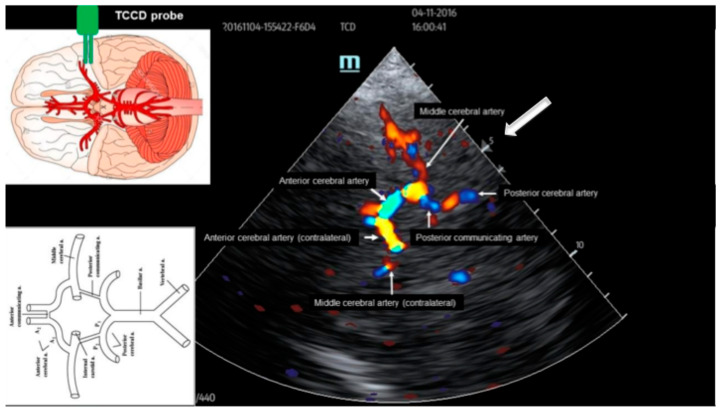
Anatomical orientation during transcranial colour-coded assessment of the circle of Willis. TCCD indicates transcranial colour-coded duplex. White arrow indicates the focus of the ultrasound beam during the investigation.

The first important step in performing a TCCD test is to find the correct transducer position. It is important to mention that the marking on the transducer should always point forward. If colour coding is not yet applied, a butterfly-like pattern is first visible on the B-mode image, corresponding to the sella turcica. The circle of Willis surrounds this. For less experienced examiners, it is recommended to use colour coding (colour mode) immediately, because in this case, the vessels become immediately identifiable. With a temporal window of appropriate quality and using an appropriate technique, the image of the circle of Willis shown in Figure 2 is obtained after colour coding. In order for the ultrasound beam to be focused at the appropriate region of interest, the depth must be adjusted, if necessary, using the depth adjustment button. When examining the circle of Willis in adults, the most appropriate registrations can be achieved by setting the depth to 5 cm in adult patients (marked with a white arrow in the image). The figure also shows how colour coding helps identify individual vessel segments in the circle of Willis. Vessel segments with flow approaching the transducer are assigned the yellow-red scale, while those with flow away from the transducer are assigned the blue-green scale. From a technical point of view, especially in the initial period of the learning curve, the flow of the middle cerebral artery is the easiest to examine because this vascular segment with flow approaching the transducer has the largest vessel diameter. Therefore, in everyday clinical practice, the examination of this vascular segment has become the most widespread. The assessment of the other vascular segments forming the circle of Willis may be more difficult because of their location and narrower diameter. The image also shows that, in the case of ideal technical feasibility, not only can the vessel segments on the same side as the transducer be examined, but so can the opposite side of the vessels forming the circle of Willis. This may be important, for example, in cases where transcranial Doppler examination cannot be performed on the affected side following neurosurgical intervention or for other reasons.

After the vascular segments of the circle of Willis have been localized using the colour mode, the cursor is placed in the desired vascular segment by activating the pulsatile mode. The next step is to correct the angle of incidence of the ultrasound beam and the measurement of the flow velocity values in the desired vascular segment can begin. Table 1 summarizes the ultrasound beam focus depth at which each vascular segment can be examined, and the normal mean blood flow velocity in the different vessels.

In addition to flow velocity values, most devices also provide the systole-diastole ratio (S/D) and the pulsatility index (PI) during registration. The change in the S/D ratio may be used for monitoring the intracranial pressure (see later). The pulsatility index is a derived parameter that can be calculated using the following formula:

PI = (Systolic blood flow velocity-Diastolic blood flow velocity)
 Mean blood flow velocity

**Figure 3:**
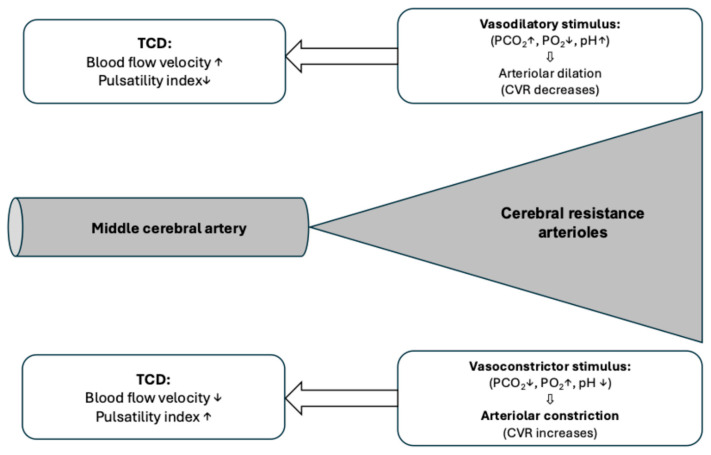
The interpretation of blood flow velocities measured using transcranial Doppler sonography. Blood flow velocity measurements are performed in the medium-sized artery, but blood flow velocity is influenced by the tone of the resistance arterioles of the corresponding vascular territory.

The normal value of the pulsatility index is close to 1, ranging normally between 0.7 and 1.2. An increase in pulsatility index indicates vasoconstriction of the resistance vessels of the given vascular territory, and a decrease indicates vasodilation of the resistance vessels. Figure 3 summarizes the changes in blood flow velocities and pulsatility indices during alteration of the different metabolic parameters (pH, arterial partial pressure of CO_2_ and O_2_). There is a non-linear relationship between PI and cerebrovascular resistance. It is positively related to the pulse amplitude of the arterial blood pressure. Additionally, it is inversely related to the cerebral perfusion pressure (CPP): the higher the CPP, the lower the PI. Thus, the determination of the pulsatility index is multifactorial: hemodynamic, metabolic changes and intracranial pressure are the key elements that may have an impact on its value.

### 1.2. Operator Dependency and Limitations of the Technique

Like all other ultrasound techniques, transcranial Doppler is an operator-dependent technique that needs substantial training and expertise. Although a recent guideline defined the basic and basic plus skills [8], it remained silent on the necessary number of investigations for gathering sufficient knowledge. It is important to emphasize that the technique involves manual, handheld positioning of the probe and thus the results of the transcranial Doppler scan may vary between different performing physicians. Additionally, the middle cerebral artery is the vascular segment that can be insonated most simply through the temporal window. Finding the location as well as measuring blood flow velocities in other vascular segments may necessitate more experienced examiners. An important further factor for inter- and intraobserver measurement variability is the angle of insonation. Using the handheld transcranial Doppler probe, no angle correction of the ultrasound beam is possible and this may influence the blood flow velocity values. As the transcranial colour-coded technique enables the visualization of the different vessel segments, it also enables the angle correction. The usability of each temporal window may vary from person to person. It should also be noted that in approximately 10–20% of people, none of the temporal windows can be used for transcranial Doppler registration due to the structure of the diploe of the skull bone [9]. Additionally, advanced age and obesity may also limit the accuracy of the temporal window.

## 2. Clinical Use of Transcranial Doppler Sonography in the Intensive Care Unit

Since the development of transcranial Doppler sonography by Aaslid in 1982 [10], the method has taken off and has been used in various disorders for monitoring cerebral circulation. Table 2 summarizes the results of the transcranial Doppler sonography studies performed in patients under critical care conditions [11].

The method contributed to a better understanding of the pathophysiological processes occurring in the cerebral circulation under critical care conditions; hence, there are only a few areas where it may be useful in making diagnostic and therapeutic decisions in daily practice. In the following, we will focus on these conditions.

## 3. Intracranial Pressure Monitoring

Raised intracranial pressure may be a frequent complication of different acute brain lesions, including traumatic brain injury, hemorrhagic or ischemic strokes. Cerebral perfusion pressure-oriented treatment has become the key to the management strategy in the past decades [12]. In traumatic brain injury, a recent consensus statement gave a strong recommendation for the use of transcranial Doppler ultrasound as a non-invasive diagnostic tool for the assessment and bedside follow-up of intracranial pressure [13].

### Informative, Basic Parameters for Daily Use

**Figure 4:**
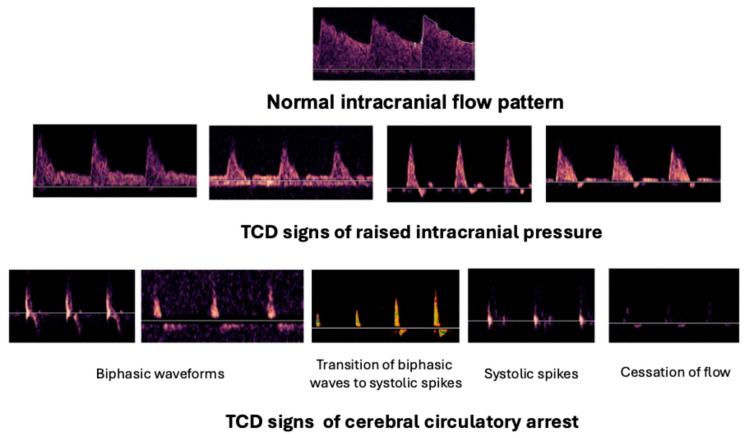
Normal intracranial flow pattern in the vessels forming the circle of Willis (**upper** picture); transcranial Doppler signs of elevated intracranial pressure (**middle** panel) and cerebral circulatory arrest (**lower** panel).

- S/D ratio: As intracranial pressure increases, characteristic changes occur in the transcranial Doppler spectrum: the systolic peak becomes sharp, and the diastolic velocity decreases. Figure 4 depicts the typical waveform alterations in parallel with the increase in the intracranial pressure. As the systolic blood flow velocity is slightly increased or remains stable during this process, and the diastolic velocity gradually decreases, the ratio between the systolic and diastolic velocity may serve as a simple trend monitor of the intracranial pressure rise. An S/D ratio above 3 may warn of an impending intracranial pressure increase. It has to be noted that the S/D ratio has a poor sensitivity for the estimation of changes in intracranial pressure, and thus it can be proposed as a rough trend-monitoring warning sign that may indicate more sophisticated invasive or non-invasive assessment of ICP.

- Pulsatility index (PI) is a non-specific parameter for the diagnosis of raised intracranial pressure. As described above, besides the intracranial pressure, other factors may also influence the PI value. Recent investigations suggested that there is a strong correlation between the pulsatility index and intracranial pressure [14,15]. The recent B-ICONIC consensus statement suggests a threshold of PI of 1.3 in conjunction with a diastolic blood flow velocity < 20 cm/sec as a threshold for considering CBF changes potentially associated with high ICP, or for excluding it [13].

## 4. Non-Invasive Estimation of Cerebral Perfusion Pressure

Calculating cerebral perfusion (CPP) pressure requires the knowledge of intracranial pressure (ICP), because CPP equals the sum of mean arterial pressure and the ICP. A sensitive method for calculating CPP by the use of transcranial Doppler sonography has been developed by Czosnyka in 1998 [16]. The non-invasive estimation of cerebral perfusion pressure is based on the following equation:

nCPP = invasive mean arterial pressure × (diastolic blood flow velocity/mean blood flow velocity) + 14.

It has been shown that invasively measured and estimated cerebral perfusion pressure strongly correlate and the method has a high (94%) positive predictive value for detecting low (<60 mmHg) CPP [17,18]. It is important to know that this method sensitively detects changes in cerebral perfusion pressure over time during the clinical course [18]. The negative predictive value for detecting nICP thresholds of 20 mmHg was above 90%, which could be further increased if the threshold of ICP was set to 22 mmHg [19]. It should be emphasized that the correlation between invasive and non-invasive cerebral perfusion pressure measurements was significantly lower in studies conducted in children (70% sensitivity and 76% specificity) [11,20].

## 5. Transcranial Doppler and TCCD as an Ancillary Test for the Determination of Brain Death

Transcranial Doppler or duplex sonography is widely used in many countries as an ancillary test to support the diagnosis of brain death. Depending on the local legal regulation, the method can be used in determining cerebral circulatory arrest, while in other countries, its results may refer to cessation of intracranial circulation, but it must be confirmed by cerebral angiography [21]. It is important to emphasize, that determination of brain death is mainly based on clinical symptoms (deep coma, absent brain stem reflexes and positive apnea test) and the ultrasound examination should be only a supplement and confirmation of the clinical findings. In comparison to digital subtraction angiography, TCD has a sensitivity of 90% and a specificity of 98% in diagnosing cessation of the intracranial circulation [22]. Insonation should be performed by an experienced neurosonologist/neurointensivist and should be documented in the memory store of the ultrasound device; alternatively, a printout of the registration should be produced and stored in the documentation. Bilateral middle cerebral arteries plus one arterial segment from the posterior circulation (vertebral arteries or basilar artery) should be insonated, and their results should be consistent. A minimum of 10 cardiac cycles should be followed during the examinations. To increase the sensitivity of the transcranial Doppler, two sets of measurements should be performed 30 min apart from each other [23]. It is required that the partial pressure of CO_2_ of the arterial blood be between normocapnic range (35–45 mmHg) and the mean arterial pressure should exceed 60–70 mmHg [21]. There are three typical waveforms that support the diagnosis of brain death:

- Biphasic (reverberating, pendular or oscillating) flow with no net anterograde flow;
- Systolic spikes;
- No signal.

These ultrasonic waveforms are shown in Figure 4. Note that an absent ultrasound signal can only be considered as an ancillary sign if the patency of the temporal window was verified before the diagnosis of brain death. In the pediatric population, a recent guideline suggested the use of TCCD as an ancillary test only in children with fused skull bones [24].

## 6. Assessment of Cerebral Vasospasm

Cerebral vasospasm occurs in 22–40% of patients after aneurysmal subarachnoid hemorrhage. The appearance of vasospasm typically occurs on the third day after bleeding, usually reaching its peak between days 10–14, and then normalizing around the 20th day. The detection of cerebral vasospasm is based upon the Hagen–Poiseuille law, describing flow changes with decreased vessel diameter. Narrowing vessel diameters in the different segments of the circle of Willis increases cerebral blood flow velocity. Comparative investigations indicated a fair agreement between cerebral blood flow velocities and angiographically detected severity of the vessels (Table 3).

It is important to emphasize that these cerebral blood flow velocities are characteristic of vasospasm in the middle cerebral arteries; however, there are no generally accepted criteria for vasospasm detection in the anterior and posterior cerebral arteries.

It has to be noted that the sensitivity and the specificity of transcranial Doppler sonography in detecting cerebral vasospasm are the best for the middle cerebral artery, and they are lower for all other segments of the circle of Willis [25]. A recent meta-analysis yielded comparable results [26]. Table 4 summarizes the results of the meta-analysis by Lysakowski et al., which demonstrates the different sensitivities and specificities of transcranial Doppler in detecting cerebral vasospasm of the different Willisian segments.

**Figure 5:**
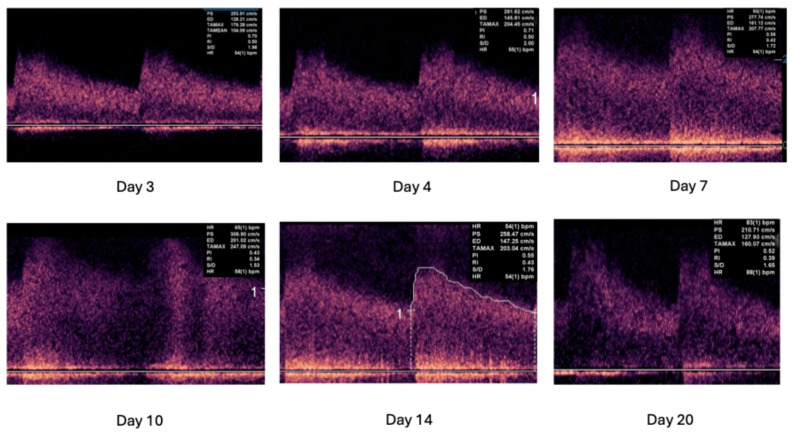
Transcranial Doppler registrations during development and resolution of cerebral vasospasm.

Figure 5 depicts serial measurement in a case of severe vasospasm after aneurysmal subarachnoid hemorrhage. Mind the upstroke and the decline of the mean blood flow velocities (TAMAX) during the course of the follow-up.

Given that the value of the flow velocity measured in intracranial vessels is also determined by factors other than the vessel diameter, Lindegaard proposed the introduction of a coefficient [27] that, in addition to the value of the intracranial blood flow velocity, also takes into account the blood flow velocity of the extracranial vessel supplying the brain (internal carotid artery).

**Figure 6:**
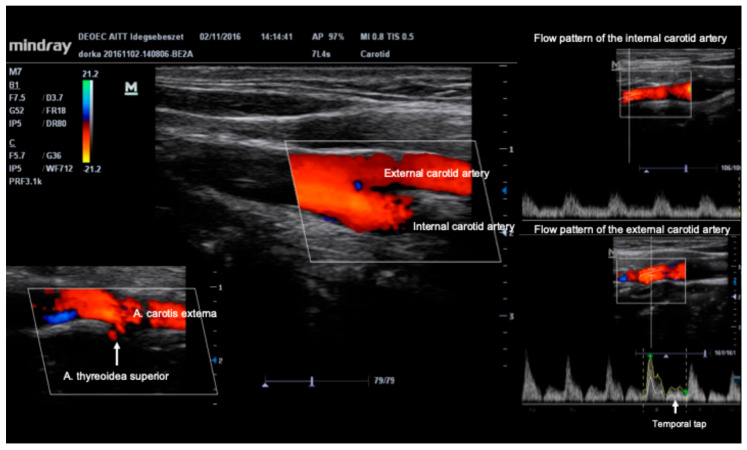
Differentiation between internal and external carotid artery using B-mode and colour-coded linear ultrasound.

The Lindegaard ratio is the ratio between the middle cerebral artery mean blood flow velocity and the mean blood flow velocity measured in the ipsilateral internal carotid artery. For the assessment of the internal carotid artery, a linear array transducer should be used with a frequency of at least 10 MHz. The differentiation of the internal and external carotid artery is possible based on the usually different widths of the two vessels (internal carotid is wider), and on the different flow patterns of the internal carotid artery (low resistance pattern) and external carotid artery (high resistance pattern). Furthermore, there is a specific anatomical feature, namely that the internal carotid artery never branches before entering the skull, while the external carotid artery always branches. In case of doubt, tapping the superficial temporal artery should be performed. This tapping appears on the Doppler spectrum of the external carotid artery, while it does not on the spectrum of the internal carotid artery. Figure 6 demonstrates the main points of differentiation between internal and external carotid arteries.

The Lindegaard ratio is considered normal if it is below 3 and a ratio above 6 indicates severe vasospasm of the vessel. Table 5 summarizes the severity of cerebral vasospasm according to mean blood flow velocity and the Lindegaard ratio.

According to a very recent meta-analysis, the highest sensitivity (0.86, 95% CI 0.71–0.94) and specificity (0.75, 95% CI 0.52–0.94) for vasospasm and delayed cerebral ischemia were achieved with a mean cerebral blood flow velocity of 120 cm/s combined with an elevated Lindegaard ratio [28]. In the pediatric population, the role of transcranial Doppler and duplex for the detection of cerebral vasospasm is a debated issue, because there is a lack of validated pediatric thresholds, and the majority of the studies applied adult-based criteria [24,29].

**Figure 7:**
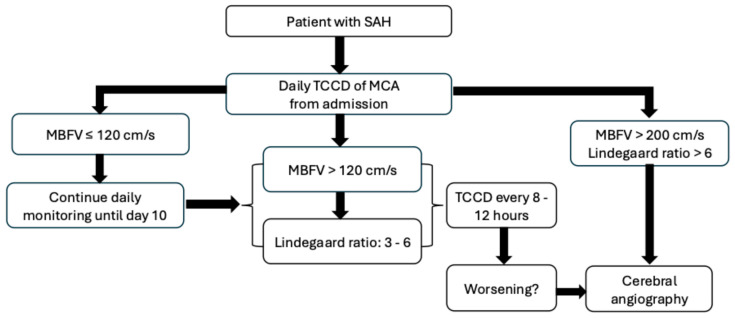
Algorithm for the use of transcranial colour-coded duplex (TCCD) in the detection and follow-up of cerebral vasospasm. MBFV indicates mean blood flow velocity; MCA indicates middle cerebral artery.

It has to be noted again that transcranial Doppler sonography does not directly measure cerebral blood flow; blood flow velocity changes are only proportional to changes in cerebral blood flow. It is also worth mentioning again that the highest sensitivity for detecting cerebral vasospasm was demonstrated in the middle cerebral artery, and in the other Willisian segments, the sensitivity of vasospasm detection is lower. Third, delayed cerebral ischemic lesions do not necessarily appear in the territories of the vessels, where cerebral vasospasm can be detected. Transcranial Doppler is most useful when viewed as a bedside, non-invasive trend monitor that can alert to the risk of developing vasospasm, and is suitable for monitoring changes in blood flow. Figure 7 provides an algorithm for the use of TCCD in detection and monitoring of cerebral vasospasm.

## 7. Future Directions of the Technique

Although the present review focuses on the basic application of TCCD in intensive care units, we have to mention the possible future directions of the technique. Artificial intelligence enables the use of automated insonation devices. These are usually multi-gated arrays that help to overcome the operator dependency of the traditional transcranial Doppler technique. They also allow continuous monitoring of the cerebral blood flow velocities as a part of a multimodal monitoring system and may be used in automated decision-making algorithms, guiding the clinicians in making rapid decisions [30]. In all fairness, these systems, although available on the market, are not yet part of the routine monitoring even in the specialized neurocritical care units.

In conclusion: TCD is a simple, yet skilled, bedside method for assessing intracranial circulation. In everyday practice, it can be used to support clinical decision-making, primarily in the areas of intracranial pressure monitoring, diagnosis and follow-up of vasospasm, and diagnosis of cerebral circulatory arrest. The study of cerebral hemodynamics should be an integral part of the increasingly widespread bedside ultrasound diagnostics in intensive care.

Of course, like all ultrasound methods, this one has its own learning curve. It is not the goal that a general intensive care physician will be able to perform all of the sophisticated applications of the method (vasoreactivity and autoregulation tests). The European Society of Intensive Care Medicine currently recommends acquiring basic competence in transcranial Doppler examinations [31].

## Figures and Tables

**Table 1:** Site of insonation, depth of ultrasound beam and normal values of the different intracranial vessels.

Window	Artery	Depth (mm)	Mean Velocity (cm/sec)
Transtemporal	Middle Cerebral (MCA)	30–67	62 +/− 12
	Anterior Cerebral (ACA)	60–80	50 +/− 11
	Terminal Internal Carotid (IC)	60–67	39 +/− 9
	Posterior cerebral (PCA)	55–80	39 +/− 10
Transorbital	Ophthalmic	40–60	21 +/− 5
	Internal carotid (syphon)	60–80	47 +/− 10
Suboccipital	Vertebral (VA)	40–85	38 +/− 10
	Basilar (BA)	>80	41 +/− 10

**Table 2:** The use of transcranial Doppler under critical care conditions.

Critical Care Condition	MBFV	PI
TBI/elevated ICP	↓	↑
Vasospasm	↑	↓
Brainstem death	↓ until 0-flow	↑
Meningitis	↑	↑
Loss of cerebral autoregulation upper threshold	↑	↑
Loss of cerebral autoregulation lower threshold	↓	↓
Severe preeclampsia/eclampsia	↑	-
Severe sepsis, septic encephalopathy	↓	↑
Fulminant hepatic failure	↓	↑
Sickle cell anemia	↑	↓
Decreased cardiac output	↓	↑
Shock, above the threshold of autoregulation	↓	↑
PaCO_2_↑	↑	↓
PaCO_2_↓	↓	↑
Hypothermia	↓	↑
Rewarming after hypothermia	↑	↓
Hypermetabolism/fever	↑	↓
Anesthetic induction agents/sedato-hypnotics	↓	-
Volatile anesthetic agents (MAC-dependent)	- or ↑	- or ↓

**Table 3:** Correlation between mean blood flow velocity and angiographically assessed severity of cerebral vasospasm.

TCD Mean Blood Flow Velocity (cm/s)	Angiographic Vessel Narrowing (%)
<125	<25%
120–200	25–50%
>200	>50%

**Table 4:** Sensitivity and specificity of transcranial Doppler sonography in detecting cerebral vasospasm compared to the gold standard angiography [25].

	Sensitivity	Specificity	Positive Predictive Value	Negative Predictive Value
MCA	67%	99%	97%	78%
ACA	42%	76%	56%	69%
PCA	48%	69%	37%	78%
BA	77%	79%	63%	88%
VA	44%	88%	54%	82%

**Table 5:** Grading the severity of the vasospasm according to mean blood flow velocity and Lindegaard ratio values.

Severity of Vasospasm	Middle Cerebral Artery Mean Blood Flow Velocity	Lindegaard Ratio
Mild	<120	<3
Moderate	120–200	3–6
Severe	>200	>6

## Data Availability

The original contributions presented in this study are included in the article. Further inquiries can be directed to the corresponding author. The authors have reviewed and edited the output and take full responsibility for the content of this publication.
